# Antibacterial Activity of *Onopordum Espinae*: Identification of Hispidulin and Dehydromelitensin-8-(4ꞌ-Hydroxy-Methacrylate)

**Published:** 2017

**Authors:** Fatma Njeh, Dhekra Mhalla, Ibtissem Ben Hammouda, Mohamed Trigui, Raoudha Mezghani-Jarraya

**Affiliations:** a *Laboratory of Organic Chemistry LR17ES08, Natural Substances Chemistry Team, Faculty of Sciences, 3000 Sfax, University of Sfax, Tunisia.*; b *Laboratory of Biopesticides, Center of Biotechnology, 3018 Sfax, University of Sfax, Tunisia.*

**Keywords:** total flavonoid content, total phenolic content, DPPH radical-scavenging assay, hispidulin; dehydromelitensin-8-(4ꞌ-hydroxy-methacrylate), antibacterial activity

## Abstract

Hexane, ethyl acetate and methanol extracts of *Onopordum espinae* leaves were investigated for their antibacterial activities against *Bacillus cereus*, *Staphylococcus aureus*, *Enterococcus faecalis*, *Escherichia coli*, *Pseudomonas aeruginosa,* and *Salmonella enteritidis*. Hexane and ethyl acetate extracts were toxic to Gram-positive bacteria with inhibition zone diameters ranging between 12 and 30 mm. Two products, hispidulin **1**, and dehydromelitensin-8-(4ꞌ-hydroxy-methacrylate) 2 were isolated from the ethyl acetate extract of leaves and identified by 2D-NMR for the first time from this species. Ethyl acetate extractꞌs total flavonoid content was the highest, as 78.73 mg quercetin equivalents (QE)/g. The methanol extract showed the highest total phenolic content as 243.43 mg gallic acid equivalents (GAE)/g and gave the most important 2,2-diphenyl-1-picrylhydrazyl (DPPH)scavenging activity (EC_50_ = 86 µg/mL).

## Introduction

Bacteria continue developing expanding defense against recent antibiotics manufactured lately by pharmaceutical companies. They become capable of gaining resistance to drugs that are used as curative agents. This is an interesting matter because of the large number of patients who have lost immunity and as a result of new bacterial strains multidrug-resistant. Therefore, new diseases can appear in hospitals resulting in high fatality.

Plants have always been an important source of natural phytochemicals for preserving health of humans, with more concern for natural therapies (1). 

Some species of the genus *Onopordum* were used as antipyretic agents and to treat diabetes, hepatic diseases, gastric disorders and gynecological diseases (2-4). Others exhibited antimicrobial and antioxidant activities (5, 6). *Onopordum espinae* Bonnet (*Asteraceae*) locally known as "Bokk" is a cotton thistle endemic to Tunisia and Libya (7). 

There are no previous studies on the chemical composition or on the antibacterial activity of this plant. Besides, there is no information about its traditional uses.

Thus, this study was carried out with the aim of evaluating the free radical-scavenging properties and the antibacterial activity of this plant, as well as identifying natural chemicals from it.

## Experimental

*Chemicals*

2,2-diphenyl-1-picrylhydrazyl (DPPH), Butyl Hydroxy Toluene (BHT), α-tocopherol and gallic acid reagents were purchased from Sigma (Germany). Aluminum chloride and Folin-Ciocalteu reagents were purchased from Fluka (Germany). Sodium carbonate solid was from Normapur (France). All other reagents were of analytical grade.

*Plant material*

*Onopordum espinae *was harvested in June 2013 from Sfax, Tunisia and identified by Pr. Mohamed Chaieb, a botanist at the faculty of Sciences of Sfax. The voucher specimen (LCSN121) was deposited at the Laboratory of Chemistry of Natural Substances, Faculty of Sciences, University of Sfax, Tunisia.

*Preparation of extracts*

Leaves of *Onopordum espinae* were air dried, powdered using a mechanical grinder and successively macerated using increasing polarity solvents (hexane, ethyl acetate, and methanol) during 48 h each. The extracts were filtered, concentrated using a rotary evaporator and kept at 4 °C until use.

*Isolation and identification of products 1 and 2 by NMR*

Successive purifications of the leaf ethyl acetate extract using silica gel column chromatography were performed using a gradient elution technique and led to the identification of two pure products. ^1^H and ^13^C NMR spectral data were compared to the previous reports (8, 9).

Compound 1: Yellow needles, C_16_H_12_O_6_. R_f_ 0.54 (hexane/ethyl acetate, 3:7, v/v). ^13^C NMR (100 MHz, CDCl_3_/CD_3_OD) δ 59.96 (C-1), 164.72 (C-2), 102.16 (C-3), 182.68 (C-4), 153.06 (C-5), 131.22 (C-6), 156.94 (C-7), 94.02 (C-8), 152.29 (C-9), 104.46 (C-10), 121.74 (C-1ꞌ), 127.93 (C-2ꞌ,6ꞌ), 115.60 (C-3ꞌ,5ꞌ), 160.81 (C-4ꞌ). ^1^H NMR (400 MHz, CDCl_3_/CD_3_OD) δ 3.90 (3H, s, OCH_3_), 6.53 (1H, s, H-3), 6.54 (1H, s, H-8), 7.79 (2H, d, 8.9 Hz, H-2ꞌ, 6ꞌ), 6.93 (2H, d, 8.9 Hz, H-3ꞌ, 5ꞌ).

Compound **2**: Yellow amorphous powder, C_19_H_22_O_6_. R_f_ 0.36 (hexane/ethyl acetate, 3:7, v/v). ^13^C NMR (100 MHz, CDCl_3_/CD_3_OD) δ 146.24 (C-1), 111.30 (C-2), 111.81 (C-3), 143.99 (C-4), 50.32 (C-5), 78.46 (C-6), 51.58 (C-7), 69.33 (C-8), 44.38 (C-9), 41.39 (C-10), 137.39 (C-11), 169.89 (C-12), 118.12 (C-13), 17.49 (C-14), 65.12 (C-15), 164.91 (C-1′), 140.08 (C-2′), 124.34 (C-3′), 60.00 (C-4′). ^1^H NMR (400 MHz, CDCl_3_/CD_3_OD) δ 5.06 (1H, dd, 17.5 Hz, 10.8 Hz, H-1), 4.23 (*α*, 1H, dd, 17.5 Hz, 0.7 Hz, H-2) 4.18 (*β*, 1H, dd, 10.8 Hz, 0.7 Hz, H-2), 4.59 (*α*, 1H, d, 0.8 Hz, H-3), 4.62 (*β*, 1H, d, 0.8 Hz, H-3), 1.73 (1H, d, 11.9 Hz, H-5), 3.69 (1H, t, H-6), 2.28 (1H, m, H-7), 4.52 (1H, m, H-8), 0.96 (*α*, 1H, dd, 2.3 Hz, 13.2 Hz, H-9), 1.18 (*β*, 1H, dd, 4.3 Hz, 13.2 Hz, H-9), 4.81 (*α*, 1H, d, 3.0 Hz, H-13), 5.26 (*β*, 1H, d, 3.0 Hz, H-13), 1.22 (3H, s, H-14), 3.16 (*α*, 1H, d, 15 Hz, H-15), 3.26 (*β*, 1H, d, 15 Hz, H-15), 5.15 (*α*, 1H, d, 1.5 Hz, H-3′), 5.49 (*β*, 1H, d, 1.5 Hz, H-3′), 3.50 

(2H, s, H-4′).

*DPPH* *radical-scavenging assay*

Free radical-scavenging activities of hexane, ethyl acetate, and methanol extracts were measured according to literature (10). The DPPH free radical-scavenging activity is based on one-electron reduction. Briefly, 1 mL of a DPPH solution (4% (w/v) in methanol was mixed with 1 mL of the plant material at varying concentrations. The reaction mixture was well shaken and incubated in the dark for 30 min at room temperature. A control sample was prepared without any plant material. The absorbance of the solution was measured at 517 nm against a blank using a spectrophotometer (JENWAY 6320, United Kingdom). Percentage of DPPH scavenged by extracts or pure products at different concentrations was calculated as follows 

% inhibition = (A_blank_ – A_sample_ **/** A_blank_) ×100

where A_blank_ is the absorbance of the DPPH control solution and A_sample_ is the absorbance of the sample. The concentration of this latter providing 50% inhibition (IC_50_) of DPPH was calculated from the graph of percentage inhibition against extract concentration. *α*-Tocopherol and BHT were used as reference DPPH - scavengers.

*Total flavonoid content*

The flavonoid content was determined based on the formation of a flavonoid–aluminum complex (11). 1 mL of the sample (containing 1 mg of each tested extract) was mixed with 1 mL of a 2% aluminum chloride methanol solution. The absorbance of the reaction mixture was measured at 415 nm after incubation for 15 min at room temperature. Quercetin was used as a standard to make the calibration curve. The amount of flavonoids was expressed as quercetin equivalents (mg QE/g dry weight of the samples). All the tests were carried out in triplicate.

*Total phenolic content*

Total phenolic content was determined by using Folin-Ciocalteu reagent (12). Briefly, 0.5 mL of the diluted sample (1 mg in 1 mL) was reacted with 0.5 mL of Folin-Ciocalteu reagent for 3 min, and then 0.5 mL of a saturated sodium carbonate solution (75 g/L) were added into the reaction mixture. Finally, 3.5 mL of water were added. The absorbance readings were taken at 725 nm after incubation at room temperature for 90 min. Gallic acid was used as a standard reference and results were expressed as milligram gallic acid equivalents (mg GAE)/g dry weight of samples. All the tests were carried out in triplicate.

*Antibacterial activity*

*Bacterial strains*

Extracts were tested against six bacterial strains from American Type Culture Collection (ATCC) : *Bacillus cereus* ATCC 14579, *Staphylococcus aureus* ATCC 25923, *Enterococcus faecalis* ATCC 29212, *Escherichia coli* ATCC 25922, *Pseudomonas aeruginosa* ATCC 27853, and *Salmonella enteritidis* (food isolate). Bacterial strains were grown on Mueller-Hinton broth (Bio-Rad, France) at 37 °C for 12–14 h. The turbidity of the overnight broth was adjusted to 0.5 McFarland standards (1-1.5 10^8 ^CFU/mL) by Densimat spectrophotometer (BioMérieux, Italy) and then diluted in Mueller-Hinton broth to a final inoculum concentration of 10^7^ CFU/mL.

*Agar well-diffusion assay*

Antibacterial tests were performed by agar well diffusion method (13) using sterile Mueller-Hinton medium (Bio-Rad, France). A fresh cell suspension (100 µL) was inoculated onto the surface of agar plates. Thereafter, 6 mm diameter wells were punched in the inoculated agar medium using sterile Pasteur pipettes and the extracts were added to each well. Negative controls consisted of 20% DMSO and 50% ethanol which were used to dissolve the plant extracts. Gentamicin (15 µg/ well) was used as a positive control to determine the sensitivity of each bacterial strain. The plate was allowed to stand for 2 h at 4 °C to permit the diffusion of the extracts followed by incubation at 37 °C for 24 h. The antibacterial activity was evaluated by measuring the inhibition zone diameter IZD (clear zone around the well) against the tested microorganisms. All tests were repeated three times. 

## Results

*Phenolic and flavonoid contents*

Total phenolic contents of the tested extracts/fraction varied from 30.5 to 243.43 mg of GAE/g of sample, using the standard curve of gallic acid (R^2^ = 0.9990). 

Using the standard curve generated by quercetin (R^2^ = 0.9999), the total flavonoid contents of extracts/fraction varied from 5.24 to 78.73 mg of QE/g of sample (Table 1).

*DPPH radical-scavenging activity*

The results of DPPH radical-scavenging activity are also summarized in Table 1. The IC_50_ values ranged from 86 to 463 µg/mL. The methanol extract exhibited the highest activity.

*Antibacterial activity*

*Onopordum espinae* extracts were tested against both Gram-positive and Gram-negative bacteria and the data are shown in Table 2. Results are given by means of IZDs. The hexane and ethyl acetate extracts showed strong antibacterial activity against Gram-positive bacteria with inhibition zones ranging between 12 and 30 mm. The methanol extract was inactive against the tested microorganisms. The antibacterial activity was observed only against Gram-positive bacteria, Gram-negative ones were resistant to the tested extracts. The best activity was exhibited by ethyl acetate extract against *Staphylococcus* *aureus*, one of the most common Gram-positive bacteria causing food poisoning, followed by *Bacillus* *cereus*.

The isolated compounds hispidulin **1** and dehydromelitensin-8-(4ꞌ-hydroxy-methacrylate) **2** were also tested for their antibacterial activities, but none of them showed any antibacterial activity against the tested bacteria within the tested concentration range (1 mg/ well)

*Structural identification of compounds 1 and 2*

**Figure 1:**
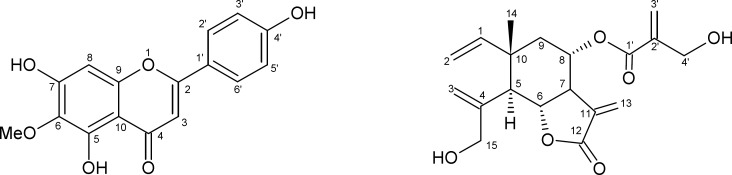
Compounds **1** and **2** isolated from *Onopordum espinae* leaves

The chemical structures of both isolated compounds were identified according to their ^1^H and ^13^C NMR data (Figure 1). Compared to literature (8-9), they were identified as hispidulin **1** and dehydromelitensin-8-(4ꞌ-hydroxy-methacrylate) **2**.

**Table 1:** Total phenolic content (TPC), total flavonoid content (TFC) and EC_50_ values of DPPH free radical scavenging of* Onopordum espinae* leaf extracts

	**Yield** **(%)**	**TPC** * **mg of GAE/g**	**TFC** † **mg of QE/g**	**DPPH** ‡ **EC**_50_ ¶ **(µg/mL)**
Samples Hex E	5.08	30.50 ± 1.1	5.24 ± 0.9	Inactive
EtOAc E	6.13	156.20 ± 0.6	78.73 ± 0.2	199 ± 1.1
MeOH E	13.23	243.43 ± 0.8	60.63 ± 0.7	86 ± 0.6
Fraction 4	-^§^	40.42 ± 0.3	24.41 ± 1.6	463 ± 0.9
1	-	-	-	310 ± 0.2
2	-	-	-	Inactive
Positive control *α*-tocopherol	**-**	**-**	**-**	26 ± 0.3
BHT	**-**	**-**	**-**	17 ± 0.2

**Hex E**: Hexane extract; **EtOAc E**: Ethyl acetate extract; **MeOH E**: Methanol extract.

* :Total phenolic content (TPC) expressed as gallic acid equivalent (mg GAE/g of tested sample).

† : Total flavonoid content (TFC) expressed as quercetin equivalent (mg QE/g of tested sample).

‡ : Results of free radical scavenging activity (DPPH assay).

¶: EC_50 _(µg/mL) values corresponding to the amount of extract/fraction required to scavenge 50% of radicals present in the reaction mixture.

§: not done.

1 : Hispidulin.

2 : **Dehydromelitensin-8-(4'-hydroxy-methacrylate)**.

**Table 2:** The antibacterial activity of *Onopordum espinae* leaf extracts

**Strains**	**Inhibition zones diameter IZD (mm)** *			
	**Hex E**	**EtOAc E**	**MeOH E**	**Genta** †
*Gram-positive*				
*Bacillus cereus ATCC 14579*	12 ± 0.5	30 ± 1.2	-^‡^	20.3 ± 0.4
*Staphylococcus aureus ATCC 25923*	12 ± 0.5	23 ± 1.1	-	25.5 ± 1.1
*Enterococcus faecalis ATCC 29212*	12 ± 0	15 ± 0.8	-	12 ± 0.2
*Gram-negative*				
*Escherichia coli ATCC 25922*	-	-	-	21 ± 1.2
*Pseudomonas aeruginosa ATCC 27853*	-	-	-	18 ± 0.6
*Salmonella enteritidis (food isolate)*	-	-	-	18 ± 0.8

Hex E: Hexane extract; EtOAc E: Ethyl acetate extract; MeOH E: Methanol extract.

Data are mean ± SD of three different experiments.

* : Diameter of inhibition zones of various extracts including the diameter of the disc (6 mm).

† : Genta: Gentamicin used as a standard antibiotic at 15 µg/ well.

‡: Activity not detected.

## Discussion

Pathogenic bacteria continue raising strong resistance and after effects against drugs, mainly antibiotics. Consequently, massive attention has been taken to extracts and biologically active compounds isolated from the vegetable kingdom. Plants can give a new source of antibacterial agents for treatment. Phenolic substances have significant antimicrobial activity and it is estimated that their function in tissues where they accumulate might provide chemical barriers to invading microorganisms (14). A wide variety of antioxidants occurs in plants, with different compositions, physical and chemical properties, mechanisms and sites of action. Several species of the genus *Onopordum* showed antimicrobial and antioxidant activities. For instance, the antimicrobial activity of *Onopordum* *cynarocephalum* Boiss and Blanche (*Asteraceae*) polar extracts was discussed (5). The antimycobacterial properties of *Onopordum anatolicum* Boiss. (*Asteraceae*) were also studied (15). *Onopordum illyricum* L. was reported to contain antioxidant compounds (6). The present work is a continuation of research in this field.

In this paper, the ethyl acetate extract of *Onopordum espinae* was the most active against Gram-positive bacteria. It was even more toxic to *Bacillus cereus* and *Enterococcus faecalis* than gentamicin, a known standard antibiotic. This significant activity is probably due to the high amount of flavonoids inside (78.73 mg QE/g), as these latter are known antibacterial agents (16). This extract was subjected to silica gel column chromatography to yield 10 fractions. The TLC profile of the fourth was the least complex; it showed two main spots with some minor compounds. Further chromatography of fraction 4 on a silica gel column too yielded 16 sub-fractions among which the sixth and the seventh were pure. NMR analysis led to identify these products as hispidulin **1** and dehydromelitensin-8-(4ꞌ-hydroxy-methacrylate) **2**.

The antibacterial activity is influenced by phenolics and flavonoids composition. A significant correlation between phenolic composition and antimicrobial activity was reported in several studies. The phenolic compounds are antibacterial agents such us gallic and ferulic acids (17). In our study, we noticed an antibacterial activity using ethyl acetate as a solvent of extraction, but the methanol extract which contains the highest concentration of phenolics (243.43 mg of GAE/g) failed to show any antibacterial activity. This result demonstrates that the antibacterial activity of *Onopordum espinae* cannot be attributed to phenolic compounds. On the other hand, flavonoids or other compounds seem to be implicated in antibacterial activity since the ethyl acetate extract contains the highest concentration of flavonoids (78.73 mg of QE/g) compared to the methanol extract. Quercetin, a known flavonoid, is an antibacterial molecule that can inhibit bacteria lipase production and inhibit D-alanine ligase activity which occurs in peptidoglycans production (18). 

In this study, the antibacterial activity was detected only against Gram-positive bacteria, no activity was observed against Gram-negative ones. These differences could be probably due to cell membrane permeability or other genetic factors. The outer membrane of Gram-negative bacteria acts as a barrier to many environmental substances including antibiotics (19). 

Hispidulin is a flavonoid identified for the first time from *Onopordum espinae*, but it was already isolated from *Onopordum* *acaulon* L., *Onopordum* *ambiguum* Fresen, *Onopordum* *corymbosum* Willk, *Onopordum* *heteracanthum* C.A.Mey. (*Asteraceae*) and others (20). Its DPPH scavenging activity of 300 µg/ mL proves its DPPH radical-scavenging effect. The free radical-scavenging assay of this flavonoid and its antibacterial activity were also described in literature (21), but methods used were different. The antifungal activity of hispidulin against *Aspergillus tubingensis*, *Botrytis cinerea* and *Penicillium digitatum* was also investigated in another report (22). These studies emphasize the efficiency of this flavonoid aglycone against bacteria and fungi as well and assert its antioxidant activity. Other medicinal properties of hispidulin such as antihepatotoxic, analgesic, anti-inflammatory, and tumor inhibiting were also reported (23-25). 

Dehydromelitensin-8-(4ꞌ-hydroxy-methacrylate) is identified for the first time from this species, but it was previously isolated from *Onopordum* *acaulon*, *Onopordum* *ambiguum*, *Onopordum* *corymbosum*, *Onopordum* *cyrenaicum* Maire & Weiller, *Onopordum* *laconicum* Heldr. & Sart. ex Rouy, *Onopordum* *leptolepis* DC. (*Asteraceae*) and others (20). This sesquiterpene lactone was not reported for any biological activity yet. In this study, it was not capable to scavenge the DPPH radical.

The methanol extract was the richest in phenolic compounds (243.43 mg of GAE/g) and was the best DPPH scavenger (86 µg/ mL). These data emphasize the role of phenolic compounds as antioxidant agents.

In summary, this study has shown that hexane and ethyl acetate extracts of *Onopordum espinae* are potentially good sources of antibacterial agents and demonstrates the importance of such a plant in medicine. The present study will be essential for selecting plant species for further investigation in the discovery of new natural bioactive substances. Further investigations should focus on the isolation and structure elucidation of antibacterial active constituents from *Onopordum espinae*.
